# Food Allergy Diagnosis in Early Childhood: Journey Mapping Study With Parents and Pediatricians

**DOI:** 10.1002/clt2.70158

**Published:** 2026-02-21

**Authors:** Madlen Hörold, Magdalena Rohr, Christian Apfelbacher, Susanne Brandstetter

**Affiliations:** ^1^ Institute of Social Medicine and Health Systems Research Medical Faculty, Otto‐von‐Guericke University Magdeburg Magdeburg Germany; ^2^ University Children's Hospital Regensburg (KUNO) University of Regensburg Hospital St. Hedwig of the Order of St. John Regensburg Germany; ^3^ Research and Development Campus Regensburg (WECARE) Hospital St. Hedwig of the Order of St. John Regensburg Germany

**Keywords:** children, diagnostic journey, health services use, prevention

## Abstract

Food allergy (FA) in early childhood can be challenging for families, even before a diagnosis is made, as parents often experience anxiety and have to change their routines. Research integrating parental and pediatrician perspectives during the pre‐diagnostic phase is scarce. This study aimed to develop a journey map illustrating the FA pre‐diagnostic process in early childhood from both perspectives. We triangulated 30 transcripts (16 parent interviews; 11 interviews and three focus groups with pediatricians) from two qualitative studies within the Food Allergy Biomarker Application Consortium (NAMIBIO App) using qualitative content analysis and data visualization. Four phases emerged: (non‐)awareness, suspicion, healthcare seeking, and diagnostics. Parents were often unaware of FA risks, even with risk factors present. Pediatricians hesitated to address FA risk proactively, due to lacking specific guideline recommendations and concerns about triggering parental health‐related anxiety. Both parents and pediatricians mentioned gaps in communication between pediatricians and midwives. During the suspicion phase, families searched for information or adjusted feeding practices while symptoms were often vague. Healthcare seeking often involved lengthy referrals. Pediatricians reported knowledge gaps among colleagues regarding FA. In the diagnostic phase, parents perceived delays in diagnosis; pediatricians mentioned limited resources, particularly for oral food challenges. Integrating both perspectives revealed shared concerns and different views on how to improve the process. Key intervention points to improve the pre‐diagnostic process include clear, up‐to‐date guidelines, risk communication and improved interdisciplinary collaboration to reduce uncertainty and promote parental confidence.

## 1 Introduction

Food allergy (FA) in children is a burden for families and the healthcare system, and may also impact children directly once they become aware of symptoms or dietary restrictions [1]. For parents, the diagnosis of a FA in their child can lead to psychosocial problems [2, 3], often due to the unpredictable risk of exposure and the practical and social burden of managing FAs in daily life [2, 3, 4, 5, 6]. For pediatricians, for example, the complex diagnostic procedures (e.g., blood tests do not provide a definitive diagnosis) and the distinction from intolerances is challenging [7].

Existing literature mainly focuses on the management of FAs or the experiences with already diagnosed FAs [4, 6]. However, there are indications that challenges often begin much earlier, and the path to diagnosis is already difficult: Focus groups with parents revealed that they underwent “a transformation journey” and “the waiting game: a period of the unknown” [4]. The cohort study of Venter et al. [8] also showed negative effects on children and their parents even before a medically confirmed FA: They compared the health status and quality of life of children with medically confirmed FA to children with suspected FA and found reduced quality of life in both groups. Yet, studies that focus on the experiences of families and describe the pathways parents take before a FA diagnosis are limited.

In addition, previous studies often lack the perspective of pediatricians. A qualitative review based on parents' experiences of raising a child with FA only drew conclusions about the role of healthcare providers, suggesting that they need to provide information and guide parents through the healthcare system [6]. To get a complete picture of the situation prior to FA diagnosis, it is important to understand both the pediatrician's perspective and the parent's experience. This study aims to describe the journey to a childhood FA diagnosis from the perspectives of families and pediatricians. Understanding the challenges and opportunities within the current healthcare system prior to FA diagnosis is essential for developing prevention strategies and improving healthcare.

## 2 Methods

### 2.1 Setting

This study was conducted within the BMBF‐funded consortium NAMIBIO app (01EA2108A‐E). The overall project aims to identify early predictors for the development of FA in children and to deliver health applications to guide healthcare professionals and parents of children at high risk for FA towards prevention and early tolerance induction.

### 2.2 Healthcare System Context

The pediatric care in Germany is delivered mostly by pediatricians, but also by general practitioners, specialist clinics (both outpatient and inpatient), as well as social pediatric centers. The situation regarding care is challenging in many places. Children's hospitals are short‐staffed. Outpatient services are inadequate, especially in rural areas [9]. Parents are free to choose their child's doctor and access specialists when available. If required, they will also be referred directly by the treating doctor. Organizations, such as the German Allergy and Asthma Association provide free educational resources about allergy prevention and risk factors and run awareness campaigns, such as “Anaphylaxis in Daycare and School.” [10, 11]. The German S3 Guideline on Allergy Prevention emphasizes breastfeeding, avoiding tobacco smoke, introducing allergens early (e.g., cooked egg and peanut for high‐risk infants), keeping indoor air clean, and timely vaccinations. The guideline discourages delaying the introduction of solid foods unless the infant is at high risk. It also acknowledges that C‐section delivery slightly increases the risk of asthma [12].

### 2.3 Study Design

We conducted a secondary analysis (SA) using interview and focus group data from qualitative studies with parents of young children [13, 14] and pediatricians. Both parents and pediatricians answered open‐ended questions about prediction and prevention of FA in children. In cases with an already existing FA, parents also shared their experiences with the pre‐diagnostic process. Thus, in this SA, which Heaton [15] describes as supra analysis (an examination of new questions that transcend the first study), we triangulated two independently collected data sets to answer a new question about the journey to childhood FA diagnosis. In contrast to the initial studies, our SA did not only focus on primary prevention, but additionally addressed secondary prevention and the pre‐diagnostic process.

A detailed description of recruitment and sampling of the primary studies can be found in the study protocol [16].

### 2.4 Analytical Framework

We used a journey mapping approach to explore processes within the health care system [17]. This method is often used to visualize the experiences that patients go through during their treatment [18, 19]. Journey mapping helps to identify key points in the care process and to understand stakeholders' perspectives, create a shared understanding, and highlight gaps in care [20]. Our journey mapping approach was inspired by Simonse et al. [21], Joseph et al. [18], and Bulto et al. [17], who define a patient journey as a comprehensible representation of a healthcare service and its processes [17, 18, 21]. The various healthcare providers that patients encounter and their interactions are part of the patient journey [17, 18, 21]. The Health Belief Model [22] and the integrated model of health literacy by Sørensen et al. [23] served as complementary theoretical frameworks for developing the journey map. Both models were necessary because they address different dimensions of the pre‐diagnostic process and allowed to understand behavioral drivers and informational challenges across the pre‐diagnostic process.

### 2.5 Participants

Participants (parents and pediatricians) for the primary studies were recruited using a multi‐stage recruitment strategy. We used snowball and theoretical sampling with the aim of capturing diverse characteristics, including gender, age, ethnicity and professional expertise [16]. The initial parents sample included three groups (parents of young children without FA, parents of young children at risk for FA, parents of young children with FA), whereas for this study only parents with a child with an existing FA were selected (see Table 1). The pediatricians sample included pediatricians working in outpatient or inpatient settings in Germany.

**TABLE 1:** Inclusion criteria.

Parents of children (0–3 years) diagnosed with FA	Pediatricians working in an inpatient or outpatient setting
Diagnosis of FA confirmed by a physician	
Living in Germany	Working in Germany
Written consent form	

Thirty transcripts, including 36 participants (16 parent interviews as well as 11 interviews and three focus groups with pediatricians), documenting the experiences up to the diagnosis of FA were included in the SA. Tables 2 and 3 show the demographics of the participants included in this SDA.

**TABLE 2:** Parents demographics.

Pseudonym (pronouns)	Age	Marital status/No. of children	Quote
Thomas (he)	Late 40s	Married, 2 children	When he was 10 months old, he ate an egg. And he went into an allergic shock. The emergency doctor had to come […].
Amy (she)	Mid 40s	Partnership, 2 children	I also told him (the pediatrician) several times that I suspected she was reacting to fish. I made sure that she was going to be tested, to make sure.
Bessie (she)	Late 30s	Married, 1 child	So, we went back to the emergency room. […] And they thought for a while whether they should use the pen. But […] they decided against it. […] The oxygen saturation was still 95, so they said: Okay, go for a walk and come back.
Cara (she)	Early 40s	Married, 1 child	The pediatrician tried to get me on the right track with cortisone, so to speak. […]. So I went back to the naturopath. […] who, of course, was against corticosteroids and said to stop everything immediately, […]. Which I did.
Holly (she)	Early 40s	Single parent, 1 child	My pediatrician suspected a cow's milk protein allergy right from the start, simply because […] this atopic dermatitis was so bad that we were hospitalized for several weeks.
Olivia (she)	Early 40s	Partnership, 1 child	[…] he started to have atopic dermatitis. […] and we even went to the hospital one weekend because we were just so desperate […] and then we got the address of […] an allergist, who looked at the skin and […] said that we would do a blood test right away.
Poppy (she)	Early 40s	Married, 1 child	My daughter has had eczema […] Then we started complementary feeding […] I gave her a porridge with hydrolyzed formula. […] The second day, I do not remember why, I gave her the porridge with the pre‐milk. Like that. She reacted […]
Rosie (she) & Michael (he)	Mid 30s/Early 40s	Partnership, 1 child	Oh God, oh God, am I even allowed to take my child into the yard? […] I think an allergy consultation would have helped me to classify that.
Sienna (she)	Mid 30s	Married, 2 children	My husband gave him some nectarines to eat and he started rubbing his eyes and they started to swell up very quickly. […]
Zelda (she)	Late 30s	Married, 1 child	Well, to be honest, not me (smiling). We didn't think about it. We were just looking forward to having our first child.
Riley (she)	Late 30s	Married, 1 child	I think when he was 7 months old, he had a reaction to cow's milk. And, well, he already had atopic dermatitis, so we suspected that he was allergic to some kind of food.
Piper (she)	Late 30s	Married, 1 child	We've never had to deal with food allergies. So at first we were a little overwhelmed and didn't know what to do.
Miranda (she)	Mid 30s	Married, 1 child	No specific recommendations. Maybe parents with pre‐existing conditions have a different experience.
Megan (she)	Early 30s	Married, 1 child	So, it took us a long time to get to the right place that could treat us properly. […]
Lola (she)	Early 30s	Married, 2 children	And then, as I said, it was quite an ordeal going from one pediatrician to the next, who somehow only wanted to prescribe cortisone. […].
Jessica (she)	Early 30s	Married, 1 child	I went into atopic dermatitis therapy with creams and lots of black tea compresses […]. It was just an endless spiral. And because it wasn't getting better, we had a blood test […].

**TABLE 3:** Pediatrician demographics.

Pseudonym (pronouns)	Age	Care setting	Quote
Camilla (she)	Early 60s	Primary care pediatrician	I must say, I also have an incredibly high breastfeeding rate. Because I think, I put a lot of effort into it. Yes, sometimes even more than the midwives.
Liam (he)	Late 40s	Primary care pediatrician	How do they get to you? Liam: Very different. From frustrated […]. To very hopeful. There is the whole range.
Dorothy (she)	Early 60s	Primary care pediatrician	I believe in the informed patient. I try to explain to all my patients, in whatever language, why and why not.
Elizabeth (she)	Late 40s	Primary care pediatrician	Elizabeth: Unfortunately, there is no cooperation [with midwifes]. […] Although I have to say that I do not feel that, the midwives make a big deal about allergies. Darcy: If it occurs later. If they are still taking care of them and they have skin problems, sometimes they put a little flea in their ears.
Darcy (she)	Late 40s	Primary care pediatrician	
Brooke (she)	Early 40s	Primary care pediatrician	[…] also the fear of doing the wrong thing. […] I still think about it three times when they are sensitized. What do I tell the parents?
Henry (he)	Early 40s	Primary care pediatrician	[…] anything with skin, any pimple, any blemish, […] you hear that quite often, I wouldn't have come if there weren't, I don't know, three pimples on my butt. That is often connected with a fear […]
Arthur (he)	Mid 40s	Primary care pediatrician	[…], does he have atopic dermatitis? […] What was the reaction? […] and if he's eaten peanuts and he's got a little […] was a little bit red, then you don't have to make a diagnostic.
Ivy (she)	Early 30	Secondary care pediatrician	[…] I think it always depends a little bit on whether the concern is justified. And, if you think, yes, the risk is increased […], then I communicate this relatively openly with them.
Archie (he)	Mid 40s	Primary care pediatrician	[…] the idea of starting late (complementary feeding) and so on still exists.
George (he)	Early 40s	Primary care pediatrician	Exactly. So if it's […] atopic eczema, no; moderate to severe atopic eczema, then I do the testing first.
David (he)	Mid 50s	Primary care pediatrician	Thomas: Yes, it is a dilemma to diagnose real allergies in the first year of life. I have to say. It's sometimes difficult to prove it clearly, I would say
Thomas(he)	Early 60s	Primary care pediatrician	
Lilian (she)	Early 50s	Primary care pediatrician	
Daniel (he)	Late 40s	Primary care pediatrician	
Samuel (he)	Mid 50s	Secondary care pediatrician	Yes, well, first, I do not want the parents to be afraid; I want them to be sure. […] we are only going to look at the things that we suspect or strongly suspect.
Catline (she)	Late 30s	Secondary care pediatrician	Blake: Now, if the mother has a nut allergy and the child has eaten nuts and has not reacted, there is really no reason to test. […] Catline: […] the history is often enough to resolve the issue and I do not need to do any more testing.
Blake (she)	Early 30s		
Charles (he)	Mid 60s	Primary care pediatrician	We don't need an allergy test, if we don't come to a medical conclusion, […] where we have to fear side effects and where we have to see if we can still achieve something good by avoiding it.
Benjamin (he)	Early 50s	Secondary care pediatrician	[…] diagnostics, […], also creates many misunderstandings […] you should never look blindly, […] with IgE testing.

### 2.6 Data Collection

The interviews and focus group discussions were conducted between March 2022 and July 2023 in person, by telephone, or via privacy‐compliant videoconferencing systems. They ranged in length from 30 to 77 min. The interview guide for the pediatrician study included the following topics: strategies for predicting and preventing FA in children; collaboration with primary and secondary care pediatricians, allergists, nutritionists, and midwives; parental questions about risk factors for FA and prevention of FA, advice on FA risk (factors). The interview guide for the parents study included questions about their information needs, information‐seeking behavior, and health care utilization related to FA risk prediction and prevention. Data collection allowed the identification of contacts with the healthcare system, but did not explicitly ask about the patient journey. Although the guiding questions had a similar thematic focus, the perspectives differed. Parents reported on their own journey, while pediatricians reflected on more general processes, rather than on their experiences as direct counterparts to the interviewed parents.

### 2.7 Data Analysis and Reporting

First, all included transcripts from the parent study and the pediatrician study were reread to check whether the statements given have the potential to answer the research question (Step 1). Next, we defined deductive major categories based on the literature [17, 18, 21] to describe healthcare contacts, symptoms, care processes and trajectories, and experiences leading up to FA diagnosis (Step 2), and coded the material (Step 3). We compiled text passages of the main categories and formed subcategories inductively on the data material (Step 4). By coding the data, a graphical representation based on 4 phases was considered. We developed a blank graphic representation covering the first year of life and recording symptoms, parental activities, and pediatricians' activities, for example. Based on this template, we created visual records of the experiences of each participating parent. Through an iterative process, we discussed the similarities, differences, and notable features of the parents' experiences, comparing them with the pediatricians' perspectives. Two authors (MH and MR) systematically refined the codebook (Step 5) through an iterative process of application, discussion, and revision to ensure consistency. In November 2024, we held a peer debriefing to discuss our findings with other qualitative researchers. It helped to reflect and reframe our perspective [24].

MAXQDA [25] was used to manage the transcripts and to support the coding of the data. We used Canva to visualize the analyzed data. The graphical representation of the journey mapping was guided by Davies et al. [26].

### 2.8 Ethics and Data Protection

We obtained ethics approval for the parent and pediatrician study from the Ethics Committee of the Otto‐von‐Guericke University Magdeburg (184/21). Participants provided written informed consent prior to participation. The participants received monetary compensation for their time and effort. All study activities were conducted in strict compliance with the European Union's General Data Protection Regulation and in accordance with the Declaration of Helsinki [27]. An independent trusted third party at the Otto‐von‐Guericke University Magdeburg managed the consent forms and stored them separately from the study data.

## 3 Results

**FIGURE 1:**
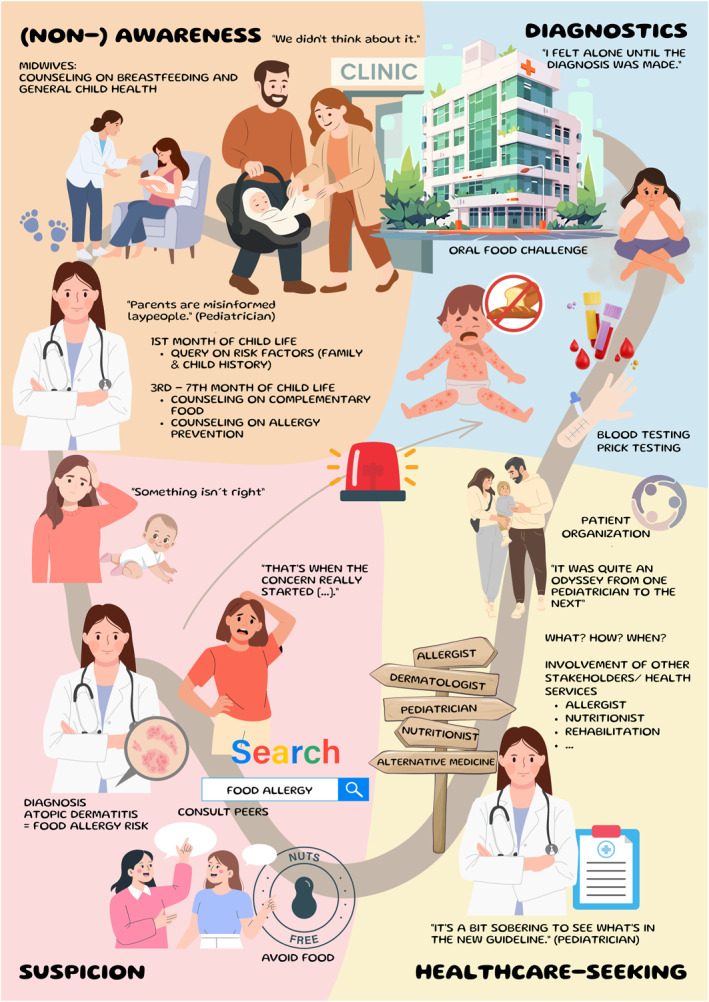
Food allergy in children—the journey to diagnosis.

We characterized four phases in the FA journey, which can vary in terms of duration: (Non‐) Awareness, suspicion, healthcare seeking and diagnostics (see Figure 1). In each phase, we identified actions and experiences as perceived and described by parents and pediatricians. Both groups reflected on their own actions and experiences and, from their perspective, on actions of other healthcare professionals (HCP) (e.g., midwives, allergists). In addition, we identified needs of parents and HCPs for each journey phase that were either directly mentioned or concluded from the interview statements.

One key finding is that parents and pediatricians did not see the whole process as a predetermined journey that they (have to) follow until a child is diagnosed with a FA. Rather, there are many intersections, or decision‐making situations. For example, this could involve implementing primary and secondary prevention measures, recognizing symptoms, and interpreting them correctly. It could also involve finding the “right” contact person in the healthcare system. Another key finding was that, although both parents and pediatricians passed through four phases, these phases varied in duration and did not necessarily occur in parallel.

### 3.1 (Non‐)Awareness

The phase of “(Non‐) Awareness” mainly refers to the period of pregnancy, childbirth and the first months of the child's life. Parents mostly had contact with midwives, obstetricians/gynecologists and pediatricians during this period. During pregnancy, parents generally did not have contact with pediatricians unless they already had older children.

In most cases, pediatricians reported that parents were not aware of FA risk factors in the period of pregnancy, childbirth and first months of the child's life. Only in some cases, parents were aware of the risk. This was in case of a FA history in the immediate family (e.g., if there was an affected sibling) or if parents themselves were affected.

We didn't think about it. We were just looking forward to having our first child. And we dealt with everything, but not really (with FA). I think, like all parents, we just hoped that it would be a healthy child. And I ate healthy during my pregnancy. But we didn't really think about it at that time. We did with the second child (smiling), but not with the first (Zelda, mother).

Pediatricians reported asking about family and child history at the first well‐child visits (at 4 weeks, 3 months and 6 months). Although there was no consensus on the right time, all pediatricians considered the medical history as an important “tool”, including the assessment of risk factors. They also mentioned giving advice on complementary feeding and allergy prevention in general. When speaking about the optimal timing to discuss allergy prevention with parents, they also pointed out the potential for early education by gynecologists and midwives. Pediatricians attributed their own uncertainty about the “right time” partly to the lack of specific recommendations for prevention in the presence of risk, but mainly to the fact that they did not want to cause unnecessary anxiety or concern among parents. Another reason for not explicitly addressing allergy risks was the large number of other topics that pediatricians felt needed to be discussed in the early months of a child's life, including vaccinations.

When is the right time to bring this up? Sometimes there's so much going on, they are excited, they are nervous (Darcy, pediatrician).

Regarding information, some pediatricians perceived “*that parents are not lay people when it comes to allergies, they're misinformed lay people. So they are definitely confronted with the issue, there are allergies. But they are misinformed. And that misinformation comes from the media, but also from the professionals* (Benjamin, pediatrician)”. Parents also expressed a need for guidance on how to find trustworthy health information and using relevant search terms, to identify credible sources, and conducting structured online searches, as they were most likely to search online for information related to children's health in general.

So of course I tried to find out a lot on the Internet. But there's always the question of what source you have in front of you (Olivia, mother).

However, parents also consulted pediatricians, midwives, and family and friends for health information regarding FA prediction and prevention.

Most parents described how they were initially unaware of the risk of FA. They criticized the fact that gynecologists and midwives did not inform them about FA prevention during pregnancy or around the time of giving birth. They did not remember that HCPs had asked them about previous family medical conditions and risk factors. “No. Actually, no one asked for that either (Zelda, mother)”. During this phase, they searched for and read information about child health in general and sometimes specifically about breastfeeding and complementary feeding, although not with a focus on prevention. “And I don't think I ever thought about it before because I don't know anyone in my social circle and there is no one in our family who has […] an allergy. And yes, that's why I didn't do any research on prevention (Jessica, mother).” “So how can you change that? […] talk, talk, talk, educate, educate and also simply educate and make sure that they understand […] (Arthur, pediatrician).” “That's why I suffer a lot when it has to be done quickly because then I don't have time for it and then I have to pay for it later because I have to explain it again the next time because they didn't have time to understand it (Dorothy, pediatrician).” Correspondingly, the pediatricians stated that the competence of colleagues in allergology should be improved, “but also by finally remunerating allergology as it should be, because it is a very important topic and also the prevention of allergies (Thomas, pediatrician)”.

### 3.2 Suspicion

We labeled the phase when parents and pediatricians notice symptoms or are concerned about unclear changes in the child's health as “suspicion”. Suspicion mainly refers to the first few months of the child's life. During this time, parents had contact mainly with midwives and pediatricians. The fixed intervals of the well‐child visits primarily determined the contact with the pediatricians. In contrast to the (non‐)awareness phase, we found that parents' and pediatricians' thinking about (food) allergy was related to concerns, suspicions, and weighing‐up signs and symptoms. We found that parents and pediatricians' considerations on the topic of (food) allergy (prediction/prevention) are not always congruent in terms of time.

Pediatricians in general assessed the risk of FA based on family and medical history; sometimes the increased risk resulted from a diagnosis of atopic dermatitis. Accordingly, the pediatricians were sensitized to the topic. We found that many pediatricians did not tell parents about the increased risk because they did not want to worry them. According to pediatricians, parents' uncertainty about symptoms often leads them to avoid certain foods. “So when it comes to prevention, exactly, I try to keep a low profile because it's so vague, and then I try, as I said, to say in general terms what I think is important for the little ones, so (Henry, pediatricians)”. As a result, parents were often not informed of the risk during the medical history process, but only became aware when the child showed symptoms or was diagnosed with atopic dermatitis. “Well, it all started when he developed severe atopic dermatitis at the age of 3 months and that was the beginning of it all […] (Jessica, mother).” “And then it is often like this, […], when they come in with atopic dermatitis, […] I always focus on skin therapy first. And I do not actually say anything about allergies if the parents do not ask of their own accord, so that they are not immediately pushed in that direction (Elizabeth, pediatrician).”

Parents talked about how they themselves became concerned that something might be wrong, for example, because they felt that the child was not eating well or they noticed changes in the child's skin. In these cases, parents took the initiative and consulted pediatricians or midwives, or sought advice from friends and family. In many cases, these situations were the starting point for seeking information (usually online) about prevention and food allergies.

Exactly, but that was the way it was. We just clearly went through Google, which somehow caused panic, it took two weeks. […] I really had one panic attack after another. And for me everything was a danger at that moment. And maybe it would have really helped if someone had classified it (Rosie & Michael, parents).

Midwives usually provided support only for the first few weeks, and some parents returned to them for the introduction of complementary feeding. Parents valued midwives' support as an important source of help and information. “Yes, of course I asked the midwife. Of course she will be there for you the first time and will continue to give you advice as long as you're breastfeeding (Sienna, mother)”. At the same time, many midwives were perceived as overworked. Pediatricians had different opinions about midwives' knowledge of FA (prevention). In some cases, midwives were criticized for providing outdated information on FA prevention and for worrying parents when risk factors were present. “I keep noticing that midwives often recommend avoiding certain things if there is a history of FA in the family, when in fact the opposite is true and in many countries it is even deliberate to include certain things in the baby's diet (Balke, pediatrician)”.

Pediatricians voiced that there was generally little cooperation with midwives. “So we hardly have the opportunity to exchange thoughts […] to meet or talk on the phone […] we work side by side. […] it is difficult to find people, to make contact. It is difficult in everyday life. […] sometimes it is not even wanted, I have the feeling. […], some people are still stuck in some things. Then it is recommended even though there is no reason to recommend it (Georg, pediatrician)”.

Consequently, pediatricians in hospitals “should work towards allergy prevention at an early stage together with the midwives, the pediatricians in primary care, and the obstetricians (Benjamin, pediatrician)”.

### 3.3 Healtcare Seeking

The phase named “healthcare seeking” covers the period when parents used health services to clarify unclear symptoms. In most cases, this occurred in the second half of the child's first year of life. During this time, parents had several contacts with different health professionals.

Pediatricians reported varying levels of knowledge within their professional group regarding the prevention and diagnosis of FA. They expressed concerns about getting it wrong, uncertainty about the appropriate timing and symptoms for allergy diagnostic testing, and whether to test for sensitization, which they often felt had no therapeutic impact. “We do not need an allergy test [in case of atopic dermatitis] if we do not draw any conclusions. We would only draw a conclusion if the clinical picture were so bad that we are now in a therapeutic dosage range of cortisone or we have to fear side effects, and we have to see whether we can still achieve something good by a nutritional leave. Otherwise, I am in favour of further exposure, and then of course you do not need to tests. (Charles, pediatrician)” “And when you do a diagnostic test in these children, you often find sensitization. That is not always clinically relevant. And then you have the dilemma of doing an oral food challenge […] So what happens? They leave everything out. And they have never had anaphylaxis before. They just get it because they have been on a diet for 5 years or something. So I'd be a little hesitant to diagnose them all at first. (Elizabeth, pediatrician).”

Depending on the care setting (primary care or hospital), the pediatricians described involving other stakeholders or being themselves involved by other pediatricians to clarify symptoms. However, they also talked about parents who, out of concern and uncertainty, contacted various health care professionals for a second opinion. “*They come prepared […] by someone. Also very popular:* “My alternative practitioner said that/.” […] Sometimes they come with blood tests (Dorothy, pediatrician)”. This was reflected in many parents' accounts. Parents consistently reported that they contacted pediatricians, allergists, dermatologists, alternative practitioners, nutritionists and patient organizations. “Well, it took us a very long time to get to the right institution that could treat us appropriately (Megan, mother).” Some parents also reported that they somehow “went to the hospital on a weekend because we were just so desperate (Olivia, mother)”.

In some cases, parents were referred to an allergist by their pediatrician, in other cases they arranged themselves additional appointments. “I made an appointment […] with our local pediatric allergist […]. And then we went there, and of course he asked to hear everything again […] (Sienna, mother).” “We actually went to a nutritionist. We even had a dietary protocol done to make sure she was not missing anything. Which was probably way over the top (Rosie & Michael, parents)”. The parents looked for explanations and a way of dealing with guilt. “So I sat with the nutritionist, who was also there at some point before the oral food challenge, and I was really in tears because I thought, did I do something wrong as a mother, so could I have done something differently? (Sienna, mother).”

Other parents reported that, initially, “we did not do anything concrete about it.” At some point, the pediatrician said that she would advise me to go to rehabilitation. Then, we went to rehabilitation, and that is when things started to happen, so to speak. That is when they started looking at the diagnosis (Cara, mother).

In this phase, too, all pediatricians shared the desire to reduce fear and uncertainty among parents. They expressed their disappointment with the current S3 guideline for allergy prevention in Germany. In their opinion, the guideline did not contain any specific options for action in the case of existing risk factors. Therefore, they could only give general recommendations to all parents. Since risk prediction has no therapeutic consequences, it was considered unnecessary.

There is no hypoallergenic infant formula. And we still have a lot of educational work to do in Germany. […] it is still in many people's minds. It is still an old myth, and that means that there will still be midwives or pediatricians who will prescribe hypoallergenic infant formula for children at risk, probably for years to come. Not everyone has realized that we have new guidelines for allergy prevention. And this is sometimes a good time to remind them, for example at U2, when mothers are discharged. But it hasn't reached all the pediatricians in private practice either (Benjamin, pediatrician).

### 3.4 Diagnostics

We named the time when FA in children is diagnosed “diagnostics” phase. During this time, the parents had contact with pediatricians, allergists, and sometimes the emergency room. The participants reported that for diagnostic purposes, it might be useful to keep a food and symptom diary for a few days to weeks. A skin test and blood test (usually for IgE antibodies) can also be helpful. Furthermore, “taking away fear is, […] something very important in allergy diagnostics.” *(Samuel, pediatrician).*

So the most important thing is that you always conduct an accurate medical history, even in the family (Arthur, pediatrician).

I would say it is the most important part of the diagnostic. Because even with diagnostics, i.e. with a skin prick test or a blood test, i.e. an IgE test, you create many misunderstandings and you should only ever search if you have a clear anamnestic suspicion. So you should never search blindly, you should never search for a needle in a haystack with an IgE test (Benjamin, pediatrician).

Even at this phase, the pediatricians seemed to worry about the right time and discussed indicators for diagnostic measures. “Elizabeth: So signs of an immediate reaction, that's clear. And severe eczema. Brooke: Yeah, I agree with that. Although the balancing act, [...] I find it difficult to categorize when eczema is moderate and when it is severe. So even if you are dealing with it all day, it's a snapshot. […] Elizabeth: Okay. You can add a third point. And if the parents insist. Because that is often the case and then it is better to do it once before they start seeing other doctors [Ärztehopping] and everybody says something different. Darcy: That is exactly what I was going to say. So if the parents put so much pressure on you that I say okay. Elizabeth: Then I would rather do that. Because the relationship of trust and I know I can do it. (Brooke, Elizabeth & Darcy, pediatricians)”. “Well, there are pediatricians who don't work that way. They take blood samples, declare every positive IgE as an allergy, and then say you just have to leave it out. And that's where we try to de‐escalate the situation (Samuel, pediatrician)”.

To confirm a FA diagnosis, an oral food challenge test is usually needed, in which small amounts of the suspected food are eaten under medical supervision. Depending on the severity of the suspected allergic reaction, the test may be done in a practice or in hospital. Pediatricians criticized that “double‐blind oral food challenge test […] do not get implemented in very many cases. This is a problem throughout Germany. This means that the demand and the reality of FA diagnostics are very far apart” (Daniel, pediatrician).” “Often it is just […] proven sensitization and then it is called an allergy test, whether it is a skin test or a lab test, and then you have to explain what it means—so what's the difference between allergy and sensitization. So not every proven sensitization means that the food can no longer be eaten (Catline, pediatrician)”.

According to the parents' experiences, the diagnosis was often made late. “Because our former pediatrician also said, oral food challenges and all that, blood tests, that doesn't help at all, what's the point? I said, people that is GUIDELINE. The official guideline for allergies says blood test and food challenge and my pediatrician says no, guideline, we do not need any of that. Just leave it out and have a look (Poppy, mother)”. In this context, pediatricians spoke about the lack of resources for oral food challenges in the inpatient sector.

Hospital stays were often a challenge for parents: “What I didn't do was add another 4 days of oral food challenges. […] And that was simply because the reaction was so severe that I didn't want my son to suffer any more. So the line was crossed, and when I realized that he was suffering, I thought to myself, 'Well, you're never going to eat a bun again, but I don't have to put your life in danger now' (Holly, mother)”.

Exceptional cases were direct emergency contacts, where the diagnosis was usually easier or quicker because the symptoms were clear. Some parents reported that there were emergency reactions and they went straight to the hospital, so in these cases there was usually almost no phase of healthcare seeking, but rather a direct diagnostic phase. “And the second time was this really severe reaction where he ate the salmon pizza. […] And then he vomited really, really violently right after he ate it. […] He could not calm down at all. He did not fall asleep at all and after about an hour and a half, […] I noticed that he was scratching himself all over. Then I took him outside and you could see he had a rash on his neck. Then we undressed him and he had hives all over his body. It was really the whole torso of his body that was covered in urticaria. And then we went to the children's hospital ourselves (Bessie, mother)”.

## 4 Discussion

### 4.1 Main Findings

Our study identified four phases in the journey to childhood FA diagnosis, based on the perspectives of parents and pediatricians: (1) (non‐)awareness, (2) suspicion, (3) healthcare seeking, and (4) diagnostics.

1. The journey often began with a phase of non‐awareness, where most parents were not familiar with FA risk factors. Pediatricians often hesitated to raise the topic, fearing to overwhelm families. Preventive guidance was rarely provided during pregnancy or early infancy, and both groups reported a lack of structured, early allergy education.
2. The suspicion phase began when ambiguous symptoms or changes in the child's health appeared. Pediatricians often recognized risk factors such as family history or atopic dermatitis but did not always communicate them directly. Parents, on the other hand, started searching for information—mostly online—which often increased their uncertainty and emotional stress. It became clear that parents and pediatricians were not always in the same phase of the journey at the same time, which led to misunderstandings and delays.
3. Families entered the healthcare seeking phase as symptoms persisted or worsened. Parents sought help from various healthcare providers, including pediatricians and midwives. Pediatricians reported uncertainty about when and how to initiate diagnostic testing. Parents described a trial‐and‐error process characterized by frustration, conflicting advice, and emotional burden.
4. Formal diagnosis usually occurred after referral to a specialist. Pediatricians emphasized the importance of clinical history and expressed concerns about over diagnosis based on sensitization alone. Parents reported delays, inconsistent diagnostic practices, and limited access to oral food challenge testing, which prolonged uncertainty and unnecessary dietary restrictions.

Overall, the diagnostic journey was described as nonlinear, emotionally charged, and characterized by fragmented communication and structural gaps. Critical points for intervention to improve the pre‐diagnostic process include earlier and clearer risk communication, improved interdisciplinary collaboration (especially with midwives), and more consistent diagnostic pathways aligned with current guidelines.

### 4.2 Strengths & Limitations

This is the first journey mapping study to combine the perspectives of parents and pediatricians to describe the diagnostic process of childhood FA. We contributed to existing studies on pediatric FA by identifying risk situations and key moments in the healthcare process with potential for improvement. The analysis is based on qualitative interviews with parents and pediatricians living in Germany. Thus, certain aspects may not reflect healthcare in other countries due to the unique nature of the German healthcare system and cultural context. As the data were originally collected with a focus on primary prevention, the journey to diagnosis was not explicitly asked about. However, interviewees whose child had a FA shared detailed insights of their experiences, allowing for a meaningful secondary analysis, where theoretical saturation was reached. A limitation is the low socio‐demographic diversity of the parents' sample. Most participating parents were highly educated and female. Still, we found a wide range of experiences even within this group, suggesting that the diagnostic journey is complex regardless of background. Future studies should include more diverse parents populations to better understand the role of social and cultural factors.

Parents and pediatricians spoke from different perspectives: parents reflected on individual experiences, while pediatricians described general patterns from clinical practice. These views are not directly comparable, but their combination helps to identify shared challenges and opportunities to improve communication and coordination.

### 4.3 Comparison of Findings With Existing Literature

While patient journey mapping has gained increasing attention in recent years, especially in adult care and chronic conditions, there is still a lack of such studies in the pediatric field. Our study contributes to this emerging field by offering one of the first structured journey maps based on both parental and pediatric perspectives.

#### 4.3.1 Pediatric Allergy Care: Challenges and Resource Constraints

One of the very few existing studies regarding pediatric conditions is a qualitative study conducted in the UK. The study on pediatric allergy care identified barriers such as long waiting times, inconsistent referral practices, and lack of reliable information [28]. These issues led to emotional distress, frustration, and a sense of helplessness and are echoed in our findings, too. Furthermore, previous studies have highlighted the limited availability of oral food challenge. A Canadian mixed‐methods study identified resource scarcity and long wait times as key barriers to oral food challenge implementation [29], while Greiwe et al. reported that 63.5% of U.S. allergists conducted five or fewer oral food challenges per month due to constraints in time and personnel [30]. Together, the studies suggest that the journey to childhood (food) allergy is not only medically complex, but can also be challenging emotionally and organizationally.

#### 4.3.2 Underlying Factors

Two mechanisms could explain these challenges. One possible explanation emerging from our data is that pediatricians perceive current guidelines on allergy prevention insufficiently specific. Clearer and more actionable guidance could reduce variability in clinical practice and strengthen collaboration between different healthcare professionals [31, 32].

Second, health literacy may be a contributing factor: While our parents were generally rather well‐educated, their experiences suggest that high education does not necessarily equate to high health literacy, particularly in the complex and emotionally charged context of allergy [33, 34]. These underscores the need for health literacy‐responsive communication from HCPs [35].

#### 4.3.3 Theoretical Background

Our findings reflect key constructs of the Health Belief Model [22]. Low perceived susceptibility and severity influenced parental behavior, while limited self‐efficacy hindered preventive actions. One pediatrician in our study referred to parents as “misinformed laypeople” when talking about their knowledge of FA prevention. This highlights the need for respectful, health literacy‐responsive communication.

Recent initiatives, such as Germany's prenatal consultation (U0), offer opportunities to integrate allergy prevention early. Embedding FA prevention as a fixed agenda item in well‐child visits (Germany: U1, immediately after birth until U9, 60th to 64th month of life) could further strengthen shared understanding between families and healthcare providers.

## 5 Conclusions

The study highlights that understanding the experiences and care pathways of families affected by pediatric FA is essential for identifying opportunities to improve both prevention and diagnostic processes. Our findings show that the FA journey often begins with a lack of awareness and continues to be shaped by uncertainty, emotional stress, and fragmented communication. To address these challenges, parents and pediatricians need access to clear, up‐to‐date information and practical, evidence‐based guidelines. Educational initiatives and public information campaigns could help improve knowledge and awareness of FA and its prevention. Strengthening communication and collaboration between healthcare professionals—particularly between pediatricians and midwives—may also help reduce conflicting messages and support parental confidence and health literacy. Future research should explore the perspectives of other healthcare professionals involved in early child and maternal care, such as midwives and gynecologists, to better understand their role in allergy prevention and early guidance.

## Author Contributions

**Madlen Hörold:** conceptualization, methodology, investigation, formal analysis, data curation, visualization, writing – original draft, writing – review and editing, validation. **Magdalena Rohr:** conceptualization, methodology, investigation, formal analysis, data curation, visualization, writing – original draft, validation, writing – review and editing. **Christian Apfelbacher:** conceptualization, methodology, supervision, project administration, funding acquisition, writing – review and editing, resources, validation. **Susanne Brandstetter:** conceptualization, methodology, supervision, project administration, funding acquisition, writing – review and editing, validation, resources.

## Funding

The Federal Ministry of Education and Research (Grants 01EA2108B/01EA2108D) funded our study.

## Ethics Statement

We obtained ethics approval for the parent and pediatrician study from the Ethics Committee of the Otto‐von‐Guericke University Magdeburg (184/21). Participants provided written informed consent prior to participation. The participants received monetary compensation for their time and effort. All study activities were conducted in strict compliance with the European Union's General Data Protection Regulation and in accordance with the Declaration of Helsinki [27]. An independent trusted third party at the Medical Faculty of the Otto‐von‐Guericke University Magdeburg managed the consent forms and stored them separately from the study data.

## Conflicts of Interest

C.A. was Grant Holder Scientific Representative of the Core Outcome Measures for Food Allergy Action (COMFA, European COST Action 18227).

## Data Availability

The data that support the findings of this study are available on request from the corresponding author. The data are not publicly available due to privacy or ethical restrictions.
